# Novel Cell Preservation Technique to Extend Bovine *In Vitro* White Blood Cell Viability

**DOI:** 10.1371/journal.pone.0140046

**Published:** 2015-10-08

**Authors:** Emilie L. Laurin, Shawn L. B. McKenna, Javier Sanchez, Horacio Bach, Juan Carlos Rodriguez-Lecompte, Marcelo Chaffer, Greg P. Keefe

**Affiliations:** 1 Department of Health Management, Atlantic Veterinary College, University of Prince Edward Island, 550 University Avenue, Charlottetown, PE, C1A 4P3, Canada; 2 Department of Medicine, Division of Infectious Diseases, University of British Columbia, Vancouver, British Columbia, V5Z 3J5, Canada; UNIFESP Federal University of São Paulo, BRAZIL

## Abstract

Although cell-mediated immunity based diagnostics can be integral assays for early detection of various diseases of dairy cows, processing of blood samples for these tests is time-sensitive, often within 24 hours of collection, to maintain white blood cell viability. Therefore, to improve utility and practicality of such assays, the objective of this study was to assess the use of a novel white blood cell preservation technology in whole bovine blood. Blood samples from ten healthy cows were each divided into an unpreserved control sample and a test sample preserved with commercially-available cell transport medium. Samples were maintained at room temperature and stimulated with the mitogens pokeweed and concanavalinA, as well as with interleukin-12 p40. Stimulation was completed on days 1, 5, and 8 post-sampling. Viability of white blood cells was assessed through interferon gamma production determined with a commercial enzyme linked immunosorbent assay. In addition, mononuclear cell viability was assessed with propidium iodide flow cytometry. Greater interferon gamma production was observed on days 5 and 8 post-collection in preserved samples, with both pokeweed and concanavalinA stimulating positive interferon gamma production on day 5 post-collection. A greater proportion of the amount of interferon gamma produced on day 1 continued to be produced on days 5 and 8 post-collection with concanavalinA stimulation (with or without interleukin 12) as compared to pokeweed stimulation. Additionally, viable mononuclear cells were still present at eight days post-collection, with a higher mean proportion detected at days 5 and 8 in all stimulated preserved samples. This practical and simple method to extend *in vitro* white blood cell viability could benefit the efficient utilization of cell-based blood tests in ruminants.

## Introduction

Early detection of a cell-mediated response to chronic infections can aid early control measures for disease [1] by more readily detecting exposed and pre-clinically affected animals when early immune responses prevail [2]. In a cell-mediated response, pro-inflammatory cytokines, such as interleukin (IL)-12, are released from antigen-presenting cells, polarizing naïve T helper cells (Th0) into Th1 cells that secrete interferon gamma (IFN-γ) and activate macrophages [2–3]. The IFN-γ assay is a cell-mediated diagnostic tool that measures, for example in Johne’s disease (*Mycobacterium avium* ssp. *paratuberculosis* [MAP]), the animal’s exposure to the organism more effectively than humoral antibody enzyme linked immunosorbent assay (ELISA) [1, 4, 5]. Traditionally, the IFN-γ assay requires processing of bovine blood samples within 24 hours of collection [6]. However, the transit time from sample collection on-farm to an appropriate laboratory can often exceed this time limit. In a study of *Mycobacterium bovis* stimulated blood, the amount of WBCs isolated decreased from fresh to two day old blood at non-room temperatures [7]. It is for this reason that cell-mediated blood tests have held to the protocol of processing samples within 24 hours of collection [7]. Furthermore, Robbe-Austerman et al. [8] recommended that whole blood kept at room temperature should be processed within 12 hours.

Non-specific mitogens can be used to stimulate up to 90% of a lymphocyte blastogenic response, eliciting IFN-γ production regardless of disease status. Commonly used mitogens, such as pokeweed (PWM) and concanavalinA (ConA), stimulate primarily B cells or T cells, respectively, and can be used for general differentiation [2, 9]. The Th1 cytokine IL-12 promotes survival and growth of Th1 immunity, sustaining efficient numbers of memory or effector Th2 cells but inhibiting the formation of Th2 immunity. This cytokine can be used synergistically with mitogens due to its immunostimulatory effects on T cells and natural killer (NK) cells to secrete IFN-γ [10–11]. Jungersen et al. [12] stressed that although viable T cells and successful antigen presentation typically occurs within eight hours post-collection, IL-12 potentiation could prolong IFN-γ secretion upon antigen stimulation of blood cells up to 24 hours [13]. In addition to assessing cell viability through IFN-γ production, the presence of viable WBCs in a blood sample can be determined via flow cytometric analysis. It is expected that *in vitro*, T cells could have a half-life of up to 2 days [6].

Therefore, the goal of our study was to assess the use of a novel blood cell preservation medium (SCSR-T™, NonInvasive Technologies) as a practical method of extending the lifespan of WBCs, *in vitro*, to enable extended sample transit time. To evaluate this, both nonspecific, mitogenic stimulation of IFN-γ production as well as flow cytometry were used to assess cell viability in preserved and unpreserved samples over time.

## Materials and Methods

### Sample collection

Animal protocols were approved by the Animal Care Committee at the University of Prince Edward Island (Protocol Number: 13–009) before commencement of the study. Ten healthy Holstein dairy cows were selected for participation from a farm in Prince Edward Island, Canada. Approximately 20 ml of blood was collected per cow by tail vein venipuncture into four heparinized vacutainer tubes (BD Vacutainer Lithium Heparin^N^, Becton Dickinson, Franklin Lakes, NJ, USA). Following collection, all samples were immediately transported in a thermos box, without cooling (as described by Jungersen et al. [12]), to the laboratory at the Atlantic Veterinary College, University of Prince Edward Island.

### Sample preparation

Immediately upon arrival at the laboratory post-collection, all vacutainer tubes of whole blood samples were pooled into 50 ml polypropylene conical tubes per cow, after which, half of the volume from each was decanted into a second 50 ml conical tube. One tube per set was kept as whole blood for an unpreserved control, while the whole blood in the other tube was preserved with 1:1 (v/v) ratio of transport medium (SCSR-T Biological Sample Preservation Medium, NonInvasive Technologies, Elkridge, MD, USA). All 20 tubes were then maintained bench-top at room temperature (21°C) in the laboratory for eight days during the study.

### Stimulation agents

Stimulation agents for IFN-γ production and release analysis in this study included the mitogen PWM (Sigma-Aldrich, St. Louis, MO, USA), the mitogen ConA (Sigma-Aldrich, St. Louis, MO, USA), recombinant bovine IL-12 p40 (Kingfisher Biotech, St. Paul, MN, USA), and a combination of ConA and IL-12 p40. Phosphate-buffered saline (PBS; pH 7.4) was used as a negative control in both preserved and unpreserved whole blood samples. Stimulation agent concentrations, as described below, were selected based on the studies of Jungersen et al. [13], Mikkelsen et al. [14], and Stabel [2]. Furthermore, results of previous mitogen titration analyses performed in our laboratory (Maritime Quality Milk, Atlantic Veterinary College, Prince Edward Island, Canada) guided our choice of dosages for PWM and ConA to avoid over-stimulation.

### Stimulation method

Methodology for transport medium use followed the foundational guidelines from NonInvasive Technologies (SCSR-T 2007 Instructions Pamphlet, www.noninvasivetech.com, accessed 19 February 2015), where the media is used primarily for human intestinal epithelial cells [15]. However, to facilitate its use for bovine whole blood, we used the following adapted protocol for the IFN-γ production and release analysis for this study. Both unpreserved and preserved samples of whole blood were stored for eight days at room-temperature, with stimulation performed on days 0 (collection day), 4, and 7, and cells harvested one day post-stimulation (on days 1, 5, and 8, respectively). Briefly, from unpreserved samples 1 ml of whole blood was added to each of five wells per cow in flat-bottom 24-well tissue culture plates (Corning Incorporated, Corning, NY, USA). From preserved samples, 2 ml of diluted whole blood in transport medium as mentioned above, was placed into five corresponding 2 ml microcentrifuge tubes per cow and centrifuged at 500 x *g* for 10 min at room temperature. The supernatant was discarded, and the remaining pellets were re-suspended with Dubelccos’ Modified Eagle Medium (Sigma-Aldrich, St. Louis, MO, USA), supplemented with 10% fetal calf serum, to complete 1 ml per tube. The suspension was transferred to each of another five wells per cow in the 24-well tissue culture plates. Then, to each respective set of two wells per cow (unpreserved whole blood and re-suspended pellet from preserved sample), the following room-temperature solutions were added, for a total of 10 wells per cow: 2 μl PBS, 10 μl (10 μg/ml) PWM, 2 μl (10 μg/ml) ConA, 4 μl (10 U/ml) IL-12 p40, and a combination of 2 μl (10 μg/ml) ConA plus 4 μl (10 U/ml) IL-12 p40. The plates were then incubated overnight (approximately 18 h) at 37°C in an atmosphere supplemented with 5% CO_2_. After incubation, the content of each well was transferred to a respective 2 ml microcentrifuge tube and centrifuged at 500 x *g* for 10 min at room temperature. The supernatants were transferred to new microcentrifuge tubes and frozen at -20°C until ELISA assessment.

### Interferon gamma analysis with ELISA

Assessment of IFN-γ concentration per sample was completed after the last stimulation day using a commercial sandwich ELISA (ID Screen Ruminant IFN-γ kit, IDVET, Montpellier, France). Procedures followed the manufacturer’s instructions, using 25 μl each of the kit’s negative and positive control (each diluted with 25 μl of the kit’s Dilution Buffer 1) and 10 μl of samples (each diluted with 90 μl of the kit’s Dilution Buffer 1). According to the procedure protocol, the test is valid if the mean optical density (OD) of the positive control is >0.5 and the ratio of the mean values of the positive and negative controls is >3. The results for each sample were interpreted as a sample to positive (S/P) ratio, or a ratio of IFN-γ concentration to the positive control using the following formula, as per the kit’s instructions: S/P (%) = [(OD activated sample–OD control sample)/(OD mean positive control–OD mean negative control)]*100. For both the preserved and unpreserved samples, the OD for the control sample was determined from the samples with only PBS added. According to this procedure protocol from the kit’s instructions, samples with S/P ratio >15% were considered positive for IFN-γ production.

### Cell viability analysis with flow cytometry

White blood cell viability was assessed via propidium iodide exclusion by flow cytometry over time [16]. After incubation of samples and removal of supernatant as described above, the remaining pellet was re-suspended in PBS to a final volume of 1 ml. Then 200 μl of each sample was transferred to respective flow cytometry tubes. To each flow tube, 5 μl (10 μg/ml) propidium iodide (Sigma-Aldrich, Co., St. Louis, MO, USA) in PBS (Thermo Scientific HyClone, Fisher Scientific Co., Ottawa, ON, Canada) and 2 ml of 1x lysis buffer (10 ml of 10x BD Pharm Lyse Stock (BD Biosciences, Mississauga, ON, Canada) with 90 ml distilled, deionized water) was added, and the tubes vortexed to immediately lyse the red blood cells (RBCs). By lysing the RBCs, the number of remaining WBCs could be accurately determined. The tubes were then incubated at room temperature in the dark for 15 min, followed by centrifugation at 200 x *g* for 5 min at room temperature. The supernatant was subsequently aspirated without disturbing the cell pellet. Then 2 ml of 1x PBS with 1% fetal bovine serum (VWR International, Mississauga, ON, Canada) and 0.1% sodium azide (preservative; Fisher Scientific Co., Ottawa, ON, Canada) was added to the pellets, which was centrifuged at 200 x *g* for 5 min at room temperature, and the supernatants discarded. The pellets were then re-suspended in 0.5 ml of 1x PBS with 2% formaldehyde (Fisher Scientific Co., Ottawa, ON, Canada) to fix the cells, which were then kept at 4°C until flow cytometric analysis after the last stimulation day.

For flow cytometry, the samples were processed through the BD FACSCalibur Flow Cytometer (BD Biosciences, Mississauga, ON, Canada). To calculate cell percentages, 10,000 events per sample were read. Propidium iodide dye is excited at 488 nm. As the dye penetrates cells with damaged membranes [16], the resultant WBC concentration after following the procedures above was gated into respective live and dead mononuclear cell (monocytes, T cells, B cells, and NK cells) percentage and live and dead polymorphonuclear cell (eosinophils, basophils, neutrophils) percentage.

### Statistical analysis

Statistical analysis was done using STATA 12 (StataCorp LP, College Station, Texas, USA) and SAS (SAS Institute Inc., Cary, North Carolina, USA). Statistical significance was set at *P* <0.05. For the outcomes defined below, two mixed regression model structures [17] with random effects at the cow, day, and preservation levels were built including any two- and three-way interaction terms of predictors as explained below. Univariable models were assessed, and any predictors with *P* <0.20 were further analyzed in multivariable models. Back-transformed marginal predictions and pairwise comparisons, as well as contrasts with Bonferroni corrections of *P-*values for multiple comparisons, were also analyzed for each model where indicated.

The first model used a log transformed S/P ratio as the outcome for IFN-γ production analysis. The model included variables for transport media (dichotomous), treatment (five categories), and day (three categories). Treatment categories included PBS (base value), PWM, ConA, ConA+IL-12 p40, and IL-12 p40. Day categories were labelled as day 1, 5, and 8 to represent the days when cells were harvested after stimulation.

The second model used percent of mononuclear cell viability as the outcome for cell viability analysis. This percent was defined as the percent of total cells counted by flow cytometry that were viable mononuclear cells. Total cells counted (equivalent to 100%) included dead and viable mononuclear cells, as well as dead and viable polymorphonuclear cells. No transformation was required for this outcome in the regression analysis. The same predictors were included as described for the first model. No other cow- or herd-level factor information was collected at the time of sampling; and therefore, no other predictors were available to be included in the models.

## Results

### Descriptive data

There were a total of 300 observations, corresponding to whole blood samples from the ten cows. Five treatments were performed on each of unpreserved and preserved samples, on each of three time periods. There were 14 missing S/P ratio results and two missing flow cytometry results due to hemolyzed samples (no pellet-serum delineation) or due to ELISA OD results that exceeded the recordable limit.

### Stimulation effect and IFN-γ production

For the 286 observations from the ten healthy cows, untransformed S/P ratios ranged from -9.8% to 468.1%, with a mean of 20.4% and a median of 1.3%. This highly right skewed outcome was therefore adjusted to be greater than zero by adding ten units to every value, followed by a log transformation for regression analysis.

Overall, in pairwise comparison analyses, PWM and ConA were significantly different in effect from no stimulation (PBS control) (*P* <0.01), but IL-12 p40 alone was never significantly different from PBS. Furthermore, there was an overall significant effect of time (day) (*P* <0.01). The three-way interaction among time (day), stimulation method, and use of transport medium was significant (*P* <0.05) (Table 1). Pokeweed had a strong stimulatory effect, particularly on day 1, but this effect in unpreserved samples was calculated with only six samples as the other four samples were over-stimulated beyond the maximum detection abilities of the ELISA. In addition, among preserved samples alone, the use of any one of the three stimulants was significantly different (*P* <0.01) from the PBS control on both days 1 and 5. Although ConA and ConA+IL-12 p40 treatments produced very similar results, only stimulation with ConA alone in preserved samples on day 5 continued to produce IFN-γ above the positive cut-point (S/P = 15%) for the ELISA kit.

**Table 1:** Means, model estimates, and *P*-values for predictors of interest, predictor interactions, and variances for random effects.

Factor or Effect		Range of Means^a^/Estimate	*P*-value
Treatment (tx)	PBS (control)	0.00^b^ (0.00^c^)	<0.01^e^
	Pokeweed	0.53–254.12 (13.32–128.26)	-
	Concanavalin A	-0.72–31.10 (11.66–41.47)	-
	Concanavalin A + Interleukin-12	-1.04–32.10 (16.58–42.67)	-
	Interleukin-12	-1.42–0.35 (0.15–0.78)	-
Preservation (tm)		0.2	<0.05
Day		-	<0.01
Interactions	tx X tm	-	<0.01
	tm X day	-	0.01
	tx X day	-	<0.01
	tx X tm X day	-	0.01
Variances	Between cow	0.07 (23.6%^d^)	<0.05
	Between day	0.01 (2.6%)	0.33
	Between preservation	0.04 (12.0%)	<0.05
	Residual (within cow)	0.18 (61.8%)	<0.01

^a^ Estimates for treatments presented as a range of means over time.

^b^ Estimates for treatments in unpreserved samples.

^c^ Estimates for treatments in preserved samples.

^d^ Proportion of total unexplained variance.

^e^ *P*-value for complete treatment factor.

Table 2 depicts the difference in S/P ratios for each stimulation treatment as compared to the control (PBS) over time (days) for unpreserved samples and samples preserved with transport media. In addition, IFN-γ production was generally higher numerically on days 5 and 8 in preserved samples as compared to unpreserved samples. However, using Bonferroni corrections, only the combination of ConA+IL-12 p40 showed statistically significant stimulation of IFN-γ production on day 8 in preserved samples as compared to unpreserved samples (*P* <0.02).

**Table 2:** Change in sample to positive ratios of interferon gamma production over time (days). On each day, this change was determined by comparing the use of a stimulant (pokeweed mitogen, PWM; concanavalin A, ConA; interleukin-12 p40 potentiated ConA, ConA+IL-12 p40; or IL-12 p40 alone) versus no stimulation (phosphate buffered saline) for white blood cells unpreserved or preserved with a transport medium (SCSR-T, NonInvasive Technologies) supplemented in whole blood samples from ten healthy cows.

	Unpreserved Blood			Preserved Blood		
	Day			Day		
	1	5	8	1	5	8
**PWM**	265.7^a^ (6^c^) [101.5–429.8]^b^	23.2^a^ (9) [7.1–39.3]	-1.3 (9) [-6.6–4.0]	95.8^a^ (10) [45.0–146.6]	19.9^a^ (10) [6.1–33.7]	6.1 (10) [-1.5–13.7]
**ConA**	24.9 (10) [8.8–41.0] ^a^	7.0 (9) [-1.5–15.6]	-1.8 (9) [-6.9–3.4]	26.8^a^ (10) [9.8–43.8]	15.9 ^a^ (10) [3.9–27.9]	5.5 (10) [-1.9–12.8]
**ConA+ IL-12 p40**	23.8^a^ (10) [8.2–39.4]	6.7 (9) [-1.7–15.1]	-3.6 (8) [-8.5–1.3]	30.3^a^ (10) [11.6–48.9]	13.5^a^ (10) [2.6–24.5]	6.9 (10) [-1.0–14.8]
**IL-12 p40**	-2.0 (10) [-7.0–2.9]	-0.4 (9) [-6.3–5.5]	0.5 (8) [-5.6–6.5]	-0.9 (10) [-6.1–4.3]	0.1 (10) [-5.7–5.9]	0.6 (10) [-5.0–6.3]

^a^ Significantly different (*P* <0.01) sample to positive ratio compared to no stimulation (phosphate buffered saline) as determined by Bonferroni corrections of *P-*values for multiple comparisons.

^b^ 95% confidence interval.

^c^ Total number of observations.

### Mononuclear cell viability

For the 298 observations from ten healthy cows, percent of viable mononuclear cells ranged from 7.7% to 81.8%, with a mean of 50.2% and median of 54.9%. No transformation was required for regression analysis.

**Fig 1:**
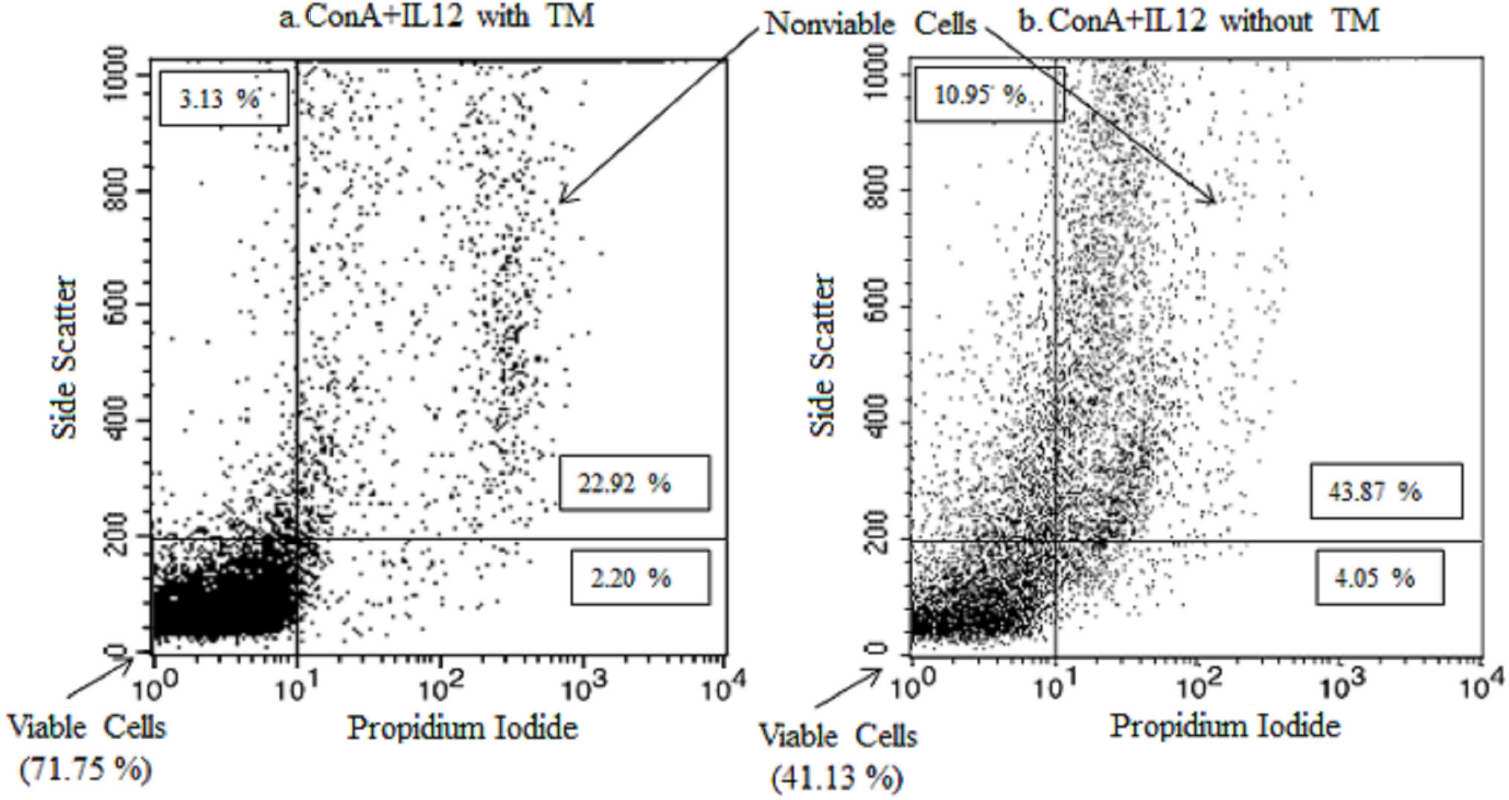
Propidium iodide flow cytometric analysis of viable and nonviable cells in preserved and unpreserved samples. Results from day 8 of the sampling period are shown for whole blood samples, either preserved (panel a.) with a transport medium (TM; SCSR-T, NonInvasive Technologies) or unpreserved (panel b.), that were collected from ten healthy cows. Arrows identify areas representing viable and nonviable cells, with the percentage of cells measured labelled per quadrant. Representative samples shown were samples stimulated with interleukin-12 p40 potentiated concanavalinA (ConA+IL-12).

Univariable regression models showed highly significant day effect (*P* <0.01) and transport medium effect (*P* <0.01) on the percentage of live mononuclear cells counted by flow cytometry (Fig 1). Stimulant use, however, had no significant effect and was subsequently not included in the multivariable model, after checking for confounding effects and model testing with and without this parameter.

**Fig 2:**
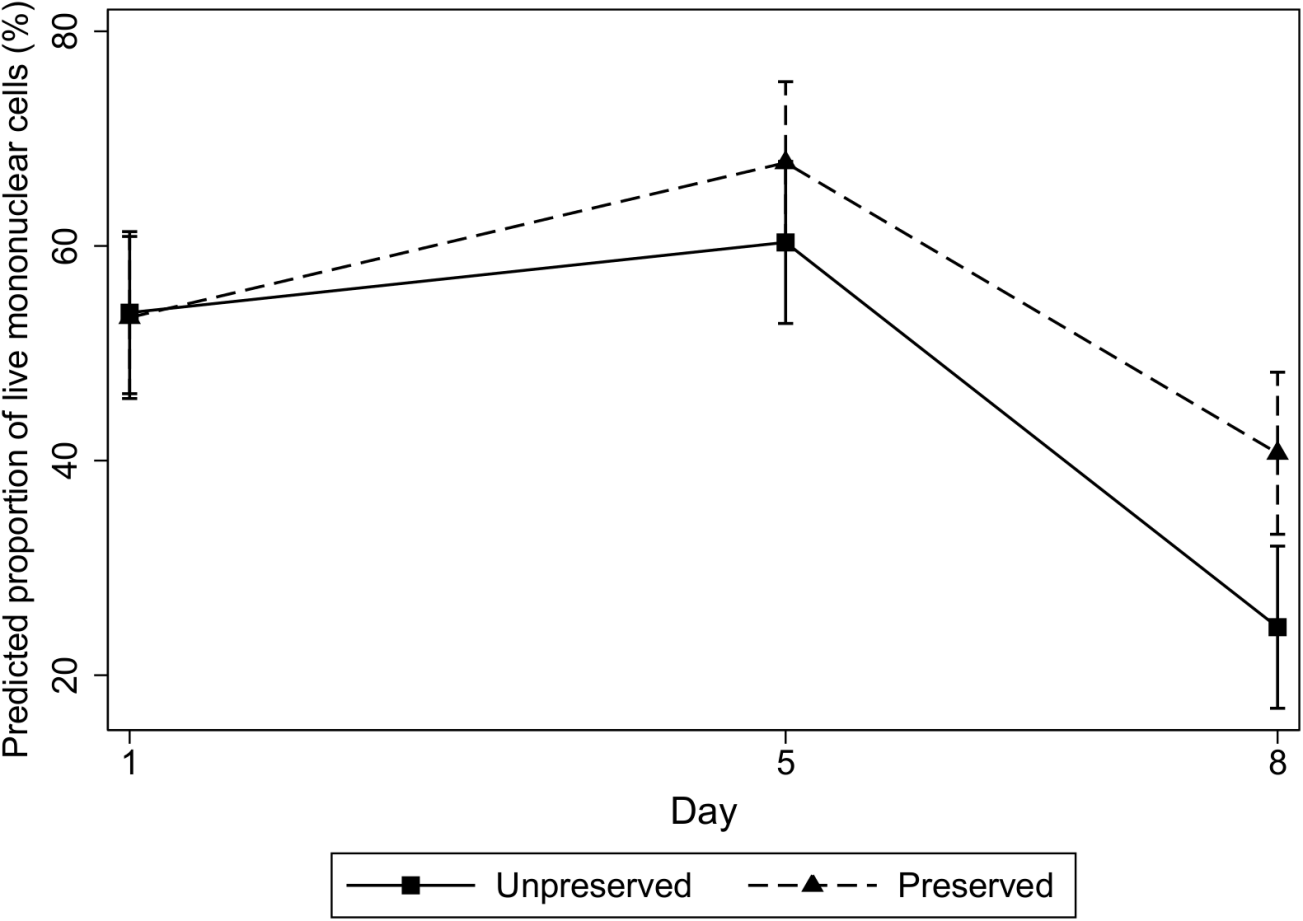
Predicted average proportion of mononuclear cell viability (with 95% confidence intervals) over time (days). Predicted results are shown for whole blood samples, either unpreserved or preserved with a transport medium (SCSR-T, NonInvasive Technologies), that were collected from ten healthy cows.

The final mixed linear multivariable regression model showed a highly significant interaction (*P* <0.01) between day and transport medium parameters. When comparing preserved and unpreserved samples (Fig 2), the proportion of mean live mononuclear cells present in unpreserved samples on day 8 was 45.3% of the mean amount present on day 1. However, in samples preserved with the transport media, 76.4% of those present on day 1 were still viable on day 8.

The mean mononuclear cell viability was 7.9% higher (95% CI: -5.9% to 21.7%) (*P* = 0.4) for preserved samples versus unpreserved samples on day 5 (as compared to day 1). This difference was 16.6% (95% CI: 2.8% to 30.5%) on day 8 (as compared to day 1) in favor of preserved samples (*P* <0.02). Within the results for day 8, significantly more viable cells were present in the preserved samples (*P* <0.01).

## Discussion/Conclusions

The application of a cell viability transport medium could allow for more efficient utilization of blood diagnostic tests, such as the cell-mediated IFN-γ assay, in veterinary applications. Our study assessed the use of the SCSR-T transport media in bovine whole blood samples. For this assessment, an evaluation was performed using non-specific mitogens, prior to evaluating the effects with specific disease antigens. Similar IFN-γ responses with mitogen-stimulated blood samples from both non-infected and sub-clinically MAP-infected cows were previously reported by Stabel [2]. Overall, our results with non-specific, mitogenic stimulation point to a potential benefit for incorporating a cell transport medium in whole blood samples to allow for longer travel times from on-farm collection to processing in an appropriate laboratory. However, further research is necessary to evaluate this benefit with the use of specific disease antigens, as different receptors, activity of antigenic stimulation on white blood cells, and host response to specific antigens could vary from the results observed in our study.

As one method for detecting cell viability over time, stimulation of IFN-γ production showed improved long-term results with preserved blood samples. Nonspecific stimulation was noted with both PWM and ConA, indicating that healthy and viable B and T cells were present over time. The benefit of the transport media for IFN-γ production in our study was observed with ConA stimulation on day 5 post-collection. Although positive effects were observed for both PWM and ConA on day 5, ConA stimulation resulted in a higher proportion of the original population of WBCs remaining healthy and viable, in comparison to results from PWM stimulation. Nonspecific mitogens can be used for positive controls in the IFN-γ assay, with a positive response to the mitogens indicating that viable and healthy immune cells are present in the sample at the time of testing [1]. Further assessment of nonspecific mitogen activity over time could assist their application as reference standards for the actions of specific antigens.

In our study, PWM overstimulated IFN-γ expression on day 1 post-collection, particularly for unpreserved samples. This overstimulation with PWM has been documented in the literature [2, 6], where some of this effect may be due to the action of PWM on primarily B cells in addition to T cells [2, 9], indicating in our study a greater presence of B cells on day 1. In contrast, ConA acts solely on T cells [2, 9]. Stabel [2] suggested that ConA would be a better stimulation agent in cell-mediated assays for chronic diseases, such as for samples from preclinical Johne’s diseased animals. Stabel [2] had also observed an increased IFN-γ production by non-stimulated WBCs after two incubation days, suggesting that this occurrence could stem from a possible spontaneous secretion of some T cell activating factors from the mononuclear cells *in vitro*.

It was difficult to accurately compare the results of our study to others analyzing the effects of mitogens, as there is no standard concentration and incubation methods throughout the literature. However, in an effort to maintain some similarity, we used the more frequently reported dosage of 10 μg/ml for each of the mitogens [2, 8], even though previous work in our laboratory (Maritime Quality Milk, Atlantic Veterinary College, Prince Edward Island, Canada) showed that a lower dosage of 5 μg/ml may also be generally effective. In contrast, a previous veterinary clinical immunology study suggested an optimal concentration of 15 μg/ml for ConA and only 5 μg/ml for PWM [18].

Furthermore, many of the studies found in the literature analyzing IFN-γ production either use heparinized blood or do not report the use of heparinized tubes during blood collection. Again, to maintain some similarity, we chose to use heparinized vacutainer tubes for blood collection. However, it has been previously noted that heparin can have a lymphocytolytic effect due to a subsequent increase of free fatty acid concentration in heparinized plasma [19]. This effect may be inconsequential if lymphocytes are isolated within 48 hours of blood sample collection [19]. Despite this potential negative effect of heparin, our cell populations still showed healthy activity and viability during our study period, particularly for blood preserved with the transport medium.

Although there is little information in the literature, the pro-inflammatory cytokine IL-12 seems to act as an inductor, polarizing Th1 immune responses [3]. Specifically, IL-12 synergistically with IL-18 induces cell-mediated immunity against mycobacteria by promoting the release of IFN-γ through the activation of Th1 cells and NK cells [3, 20]. Jungersen et al. [13] recommends the addition of IL-12 within 20 hours of blood collection in order to aid in bolstering a weaker WBC response. In our study, IL-12 p40 alone was unable to elicit detectable IFN-γ production above the cut-off for a positive result, but it was able to augment the effect of ConA. Further evaluation of this effect over time with a larger sample size is recommended.

The results of our study indicated an advantage to incorporating a transport medium at time of collection. Although only a small proportion of the original population of viable mononuclear cells was still present by eight days post-collection, as determined with flow cytometry, significantly more of these cells were detected in the preserved blood samples as compared to unpreserved blood samples. Knowledge of mononuclear cell viability over time in both preserved and unpreserved whole blood samples can enhance our understanding of the temporal results expected with IFN-γ assays post-blood collection. A simple method to assess viability *in vitro* involves the utilization of propidium iodide dye [16]. In our study, we had lysed the RBCs and used gating and propidium iodide dye in the flow cytometric analysis to separate viable and non-viable mononuclear and polymorphonuclear cells. We maintained a standard of 10,000 events each time in order to accurately compare percent of viable mononuclear cells among the three time points. Other options for cell marking for use with flow cytometry include trypan blue dye [21] or specific immune markers for T cell subpopulations with Ficoll fractionation [20–21]. The use of specific markers for Th0 and Th1 cells would have allowed for the specific identification of Th1 cells over time to correlate with the observed IFN-γ production decline over time.

Furthermore, several cow-level, environmental, and laboratory factors can lead to variation in assay results within individual cows. In particular, cow-level factors include age, gender, stress, health conditions, nutrition, and pregnancy. In addition, laboratory factors can make comparison between studies difficult. Some of these factors include not only methodology and mitogen concentrations, but also incubation periods, temperature variations, type of culture media, fetal calf serum use, and presence of possible inhibitors [21].

Through examining the use of blood cell preservation with transport media in bovine whole blood samples, our study objective was to analyze the cell preservation capabilities of the media with regards to lifespan of mononuclear cells and stimulation of IFN-γ non-specifically. Our results show that cell preservation with a transport medium could allow for extended cell viability up to eight days post-collection, with optimum cell preservation effects at five days post-collection, particularly using ConA stimulation. Therefore, a potential benefit to incorporating a cell transport medium in whole blood samples was observed, which could subsequently enhance the use of IFN-γ assays following further assessment of these techniques with specific antigens in MAP- and other disease-infected animals. The benefits could extend to any assay that requires whole blood, cell-based diagnostics.
